# Comparison of multiple genotyping methods for the identification of the cancer predisposing founder mutation p.R337H in*TP53*

**DOI:** 10.1590/1678-4685-GMB-2014-0351

**Published:** 2016-06-03

**Authors:** Mariana Fitarelli-Kiehl, Gabriel S. Macedo, Rosane Paixão Schlatter, Patricia Koehler-Santos, Ursula da Silveira Matte, Patricia Ashton-Prolla, Juliana Giacomazzi

**Affiliations:** 1 Programa de Pós-Graduação em Genética e Biologia Molecular, Universidade Federal do Rio Grande do Sul (UFRGS), Porto Alegre, RS, Brazil; 2 Laboratório de Medicina Genômica, Centro de Pesquisa Experimental, Hospital de Clínicas de Porto Alegre (HCPA), Porto Alegre, RS, Brazil; 3 Grupo de Pesquisa e Pós-Graduação (GPPG), Hospital de Clínicas de Porto Alegre, and Programa de Pós Graduação em Cardiologia, UFRGS Porto Alegre, RS, Brazil; 4 Unidade de Análises Moleculares e de Proteínas (UAMP), Centro de Pesquisa Experimental, Hospital de Clínicas de Porto Alegre, Porto Alegre, RS, Brazil; 5 Departamento de Genética, Universidade Federal do Rio Grande do Sul (UFRGS), Porto Alegre, RS, Brazil

**Keywords:** *TP53*-p.R337H, RFLP, TaqMan, HRM, Sanger Sequencing

## Abstract

Germline mutations in the *TP53* gene are associated with Li-Fraumeni and Li-Fraumeni-Like Syndromes, characterized by increased predisposition to early-onset cancers. In Brazil, the prevalence of the *TP53*-p.R337H germline mutation is exceedingly high in the general population and in cancer-affected patients, probably as result of a founder effect. Several genotyping methods are used for the molecular diagnosis of LFS/LFL, however Sanger sequencing is still considered the gold standard. We compared performance, cost and turnaround time of Sanger sequencing, PCR-RFLP, TaqMan-PCR and HRM in the p.R337H genotyping. The performance was determined by analysis of 95 genomic DNA samples and results were 100% concordant for all methods. Sequencing was the most expensive method followed by TaqMan-PCR, PCR-RFLP and HRM. The overall cost of HRM increased with the prevalence of positive samples, since confirmatory sequencing must be performed when a sample shows an abnormal melting profile, but remained lower than all other methods when the mutation prevalence was less than 2.5%. Sequencing had the highest throughput and the longest turnaround time, while TaqMan-PCR showed the lowest turnaround and hands-on times. All methodologies studied are suitable for the detection of p.R337H and the choice will depend on the application and clinical scenario.

## Introduction

Li-Fraumeni and Li-Fraumeni-Like Syndromes (LFS/LFL; OMIM# 151623) are autosomal dominant disorders characterized by increased predisposition to multiple early-onset cancers caused by germline mutations in the *TP53* gene (Malkin *et al.*, 1990). In Europe and North America, germline *TP53* mutations occur in approximately 1 in 5,000 live births (Lalloo *et al.*, 2006; Gonzalez *et al.*, 2009). In Brazil, a specific germline *TP53* mutation, p.R337H (c.1010G > A; exon 10, also known as p. Arg337His), has been described at high frequency not only in the general population of southern Brazil but also in different cohorts of patients with cancer. Carrier frequencies of 1:300 have been reported in the Brazilian States of Paraná (newborn screening program) and Rio Grande do Sul (women enrolled in a breast cancer screening cohort) (Palmero *et al.*, 2008; Custodio *et al.*, 2013). Among breast cancer-affected women unselected by family history of cancer, p.R337H has been described at a frequency of up to 8.6%, and reached 12.1% in women diagnosed with breast cancer at or before age 45 (Giacomazzi *et al.*, 2013; Cury *et al.*, 2014; Giacomazzi *et al.*, 2014). In children with adrenocortical or choroid plexus carcinomas, the same alteration has been reported at a frequency of 90% (Ribeiro *et al.*, 2001; Achatz *et al.*, 2007; Seidinger *et al.*, 2011). Thus, the prevalence of this mutation in the general population and in cancer-affected patients in Brazil is exceedingly high, probably due to a founder effect (Garritano *et al.*, 2010), classifying it as the most common germline *TP53* mutation ever described in any population. In addition to compulsory testing for the mutation in the State of Paraná since 2005, some investigators have suggested that any woman diagnosed with premenopausal breast cancer (especially when associated with a positive family history of breast cancer) in southern Brazil should be screened for p.R337H (Garritano *et al.*, 2010; Euhus and Robinson, 2013; Giacomazzi *et al.*, 2014).

Several genotyping methods have been proposed and are routinely used in clinical practice for the molecular diagnosis of LFS and LFL. Gene sequencing, however, is still considered the gold standard diagnostic method for identification of germline mutations in genes with high allelic heterogeneity, such as *TP53*. However, it is still relatively expensive and laborious and requires extensive automation, instrumentation and data interpretation. Thus, to interrogate the presence of a single mutation, alternative and less expensive site-specific testing strategies could be used. Among these, PCR-RFLP (Polymerase Chain Reaction followed by Restriction Fragment Length Polymorphism analysis), a traditional genotyping method, requires that the sequence variation under study generates or abolishes a restriction enzyme recognition site (Narayanan, 1991). After PCR amplification, the resulting DNA fragment is digested by one or more specific endonucleases that recognize restriction sites, resulting in fragments of different sizes that are then resolved by gel electrophoresis. Although this technique does not require sophisticated instruments, it is laborious and fully manual, which limits the number of analyses that can be performed in each experiment.

Allelic discrimination using TaqMan-PCR, is another mutation-specific diagnostic method which combines real-time PCR amplification and detection into a single step. Each TaqMan genotyping assay consists of two allele-specific TaqMan minor groove binding (MGB) probes containing distinct fluorescent dyes and a PCR primer pair for amplifying the sequence of interest. Cleavage of the fluorogenic probes during amplification liberates reporter dyes and its fluorescent signals indicate the allele(s) present in each sample (Livak 1999). Finally, high-resolution DNA melting analysis (HRM) was introduced in the early 2000's as a simple and inexpensive method for genotyping and mutation scanning (Wittwer *et al.*, 2003). HRM is a closed-tube mutation screening method that requires no post-PCR processing of the samples and uses specific saturation dyes that fluoresce only in the presence of double stranded DNA. After real-time PCR amplification, the fragment's melting pattern is generated by monitoring the fluorescence over a temperature range. Homozygous, heterozygous and wild type samples are distinguished according to their melting profile and melting temperatures (T_m_).

In contrast with PCR-RFLP and TaqMan assays, however, high resolution melting (HRM) is a screening method which interrogates mutations in a given PCR-amplified DNA region, but it does not allow, in most cases, precise identification of the mutation. Hence, a second, confirmation step such as DNA sequencing is required for definitive mutation diagnosis (Reed *et al.*, 2007).

In this study, we compare the performance and cost of these four different diagnostic approaches in the identification of the founder Brazilian mutation *TP53*-p.R337H.

## Material and Methods

### Subjects

DNA samples from 95 p.R337H carriers and non-carriers identified in previous research studies from our laboratory (IRB protocols 08-022 and 08-080, GPPG/HCPA), were included in this study. All individuals had consented to *TP53* genotyping for diagnostic purposes and signed an informed consent.

### DNA isolation and quantification

Genomic DNA was isolated from 200 μL whole blood using the lllustra™ Blood GenomicPrep Mini Spin Kit (GE Healthcare, UK), according to the manufacturer's instructions. DNA concentration and purity were determined using a NanoDrop 1000 spectrophotometer (Thermo Scientific, USA).

### *TP53*-p.R337H genotyping

#### Sanger DNA Sequencing

*TP53* exon 10 sequencing involves PCR amplification of exon 10, purification of PCR fragments, cycle sequencing and purification of sequencing products. PCR was performed using primers and conditions previously described (primer sequences and PCR conditions are available at http://p53.iarc.fr/Download/TP53_DirectSequencing_IARC.pdf; Petitjean *et al.*, 2007) and then treated with 10 U of Exonuclease I and 0.5 U of Shrimp Alkaline Phosphatase (Fermentas), and incubated at 37 °C for 30 min and at 80 °C for 15 min. Cycle sequencing was performed using BigDye Terminator kit version 3.1 (Applied Biosystems, USA) and the extension products were purified with BigDye XTerminator Purification Kit (Applied Biosystems, USA) according to the manufacturer's instructions. Sequencing products were analyzed on a 3500 Genetic Analyzer (Applied Biosystems, USA). Sequencing data visualization and sequence alignment were done with Chromas v2.0 and CLC Main Workbench (CLC Bio, DK) softwares, respectively.

#### PCR-RFLP assay

PCR (*TP53* exon 10) was performed according to previously published protocols (Petitjean *et al.*, 2007). PCR products were cleaved with *Hha*I at 37 °C for 2 h and then resolved in 3% agarose gels stained with GelRed™ (Biotium, USA). Resulting fragments were: 238 bp (homozygous mutant, AA genotype), 238 bp, 146 bp and 92 bp (heterozygote, GA genotype) and 146 bp and 92 bp (homozygous wild-type, GG genotype).

#### TaqMan-PCR

Custom made allele-specific TaqMan® probes were used (Applied Biosystems, USA; Assay ID TP53R337H; AHBJWZJ). Real-time PCR reactions were done in a final volume of 12.5 μL, containing 20 ηg of genomic DNA, 1X TaqMan Universal PCR Master Mix and 1X Custom TaqMan R337H Genotyping Assay. Cycling conditions were as follows: initial denaturation at 95 °C for 10 min, 40 cycles at 92 °C for 15 s and 60 °C for 1 min in a StepOne™ Real-Time PCR System (Applied Biosystems, USA). Real-Time PCR software v.2.2.2 was used for allelic discrimination.

#### High Resolution Melting (HRM)

HRM analysis was performed using a StepOne™ Real-Time PCR System according to the manufacturer's recommendations. Reactions were carried out in a final volume of 10 μL containing 20 ηg of genomic DNA, 0.3 μM of each primer and 1X MeltDoctor™ Master Mix (Applied Biosystems, USA). Primers used for exon 10 amplification were published previously (Bastien *et al.*, 2008). Cycling conditions were as follows: initial denaturation at 95 °C for 10 min, 40 cycles at 95 °C for 15 s, 57 °C for 30 s and 60 °C for 30 s. After denaturation of the PCR products at 95 °C for 10 s, HRM melting curve data were obtained by continuous fluorescence acquisition from 60 to 95 °C with at a ramp rate of 0.3%. Melting curves were analyzed with the High Resolution Melt Software v3.0.1 (Applied Biosystems, USA). Since HRM is a mutation screening method, whenever an abnormal melting curve was identified, Sanger sequencing was performed to identify the specific sequence alteration.

### Quality Control

Wild type and mutant p.R337H DNA samples, identified from previous research studies and genotyped by Sanger sequencing in two independent blood samples, were included in each run for assurance and quality control purposes (Table 1). All PCR-RFLP, TaqMan-PCR and HRM analyses were performed in duplicates and Sanger sequencing was bidirectional. All analyses were blinded with respect to the status of previous genotyping results. Three investigators reviewed all genotyping results independently.

**Table 1:** Features of each *TP53*-p.R337H genotyping method.

	Sanger Sequencing^a^	PCR-RFLP^b^	TaqMan-PCR^c^	HRM^c^
Negative controls (GG genotype)	0	1	1	3
Positive controls (GA or AA genotypes)	0	2	2	2
Patient samples tested per run^d^	47	27	22	21
Throughput (full capacity)	96	62	48	48
Total turnaround time (hours)^e^	37	13.5	3	5.25
Hands-on time (hours)	13.5	3	1	1.25

a 96-well plates

b 2 × 31-well gels

c 48-well plates.

d bidirectional sequencing, duplicate analyses for all other methods

e does not include DNA isolation and quantification.

### Cost analysis

We used the system of absorption cost analysis based on the technical protocols. Tables were set out in Excel software considering consumables, costs with laboratory personnel, direct and indirect costs associated with the laboratory infrastructure, and losses defined *a priori* at 10% (Mahony *et al.*, 2009). The costs of consumables were calculated including reagents and supplies, according to updated prices, in local currency (Gonçalves *et al.*, 2009). Personnel-related costs included estimates of labor hours and salary-related taxes in Brazil. Indirect costs were estimated through the Management Information System (Business Intelligence) of the institution. They included indirect labor costs (employee benefits including occupational medical care), air conditioning, cleaning, building maintenance, security, elevator and electrical power (Ferreira-Da-Silva *et al.*, 2012). Costs were calculated considering 100% use of the installed capacity per run for each of the genotyping techniques performed, according to the equipment available (Table 1). We have not considered costs of acquisition of the equipment, assuming that the infrastructure needed for all of the genotyping methods is already available in a given laboratory. Also, it is important to note that "maximum capacity" was considered for a given type (model) of equipment and may change with different equipment models (*i.e.* for HRM we have considered maximum capacity of use in a thermal cycler of 48 wells; estimates may differ if a 96-well equipment is used). For the cost analyses performed here we have used the equipment available in our center. The total cost of *TP53*-p.R337H analysis by each method includes all steps necessary to obtain results, including DNA isolation, quantification, all steps of each genotyping method and professional labor cost for sample handling and result interpretation.

**Figure 1:**
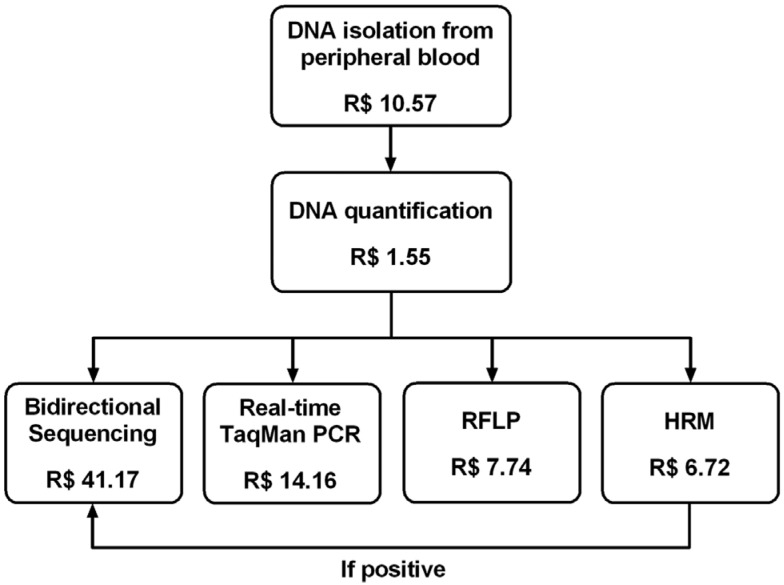
Costs of each analytical step of *TP53*-p.R337H genotyping by different methods for one patient.

Considering that HRM is a mutation screening method and direct DNA sequencing must be performed to confirm the sequence alteration if the melting profile of a given sample differs from that of WT controls, we calculated the HRM costs to analyze 100 patients, in different scenarios of mutation prevalence. For each possible mutation frequency (0 to 100%), in addition to HRM, we considered costs of Sanger sequencing of the minimal estimated proportion of samples that would have an abnormal melting profile, according to the mutation prevalence in each scenario. A single DNA isolation and quantification step was considered for each individual analyzed, as summarized in Figure 1.

### Turnaround and hands-on time analysis

To establish turnaround times, we considered the total time to perform all steps of each genotyping method using 100% of the installed capacity per run, including handling, reactions, incubations, centrifugations and interpretation of results. The hands-on time was a fraction of turnaround, comprising the hands-on steps in which an employee needs to be fully dedicated to the activity, *i.e.* sample handling and direct interpretation of the results.

## Results

To verify the performance of PCR-RFLP, TaqMan-PCR and HRM for p.R337H genotyping, genomic DNA isolated from 95 peripheral blood samples was processed by all three methods and results compared to those obtained by Sanger sequencing. Among the 95 samples analyzed, 64 non-carriers (GG genotype, wild-type homozygotes), 30 p.R337H heterozygotes (GA genotype) and 1 p.R337H homozygote (AA genotype) were identified. Results were 100% concordant with sequencing using all three methods. Representative images of results obtained with the different methodologies are shown in Figure S1.

Cost calculations for each technique were done by an administrator (RPS) and are depicted in Table 2 and Figure 1. Direct sequencing was the most expensive method followed by TaqMan-PCR, PCR-RFLP and HRM. HRM was the least expensive technology, with a cost of R$ 18.84 per patient tested, 2.83 fold less than DNA sequencing. However, it is important to note that this cost does not include the confirmatory sequencing step, needed when an abnormal melting curve is identified. The throughput of each platform used, number of controls included in each run, as well as turnaround and hands-on times needed for genotyping with all methods are summarized in Table 1.

**Table 2:** Costs of the *TP53*-p.R337H analysis for one patient.

Description	HRM^c^	PCR-RFLP	TaqMan-PCR	DNA Sequencing
Total cost (R$)^a^	18.84	19.86	26.28	53.29
Fold increase of cost^b^	1.00	1.05	1.39	2.83

a includes DNA isolation, quantification, all steps of each method and professional labor cost for handling and result interpretation

b in relation to the least expensive method

c HRM is a screening method and not a direct genotyping method as PCR-RFLP, TaqMan and Sanger sequencing (see Figure 2 for further details on additional cost).

We also assessed costs of all four genotyping strategies taking into account different scenarios of mutation prevalence. Since HRM analysis requires a sequencing step when samples show abnormal melting profiles, there is an increment of overall cost of HRM as the prevalence of positive samples increases. The other three techniques have the same overall cost for a given sample set, independent of mutation prevalence. The costs of genotyping with HRM followed by sequencing (when needed) were lower than all other methods only when the expected mutation prevalence was less than 2.5%, were equal to TaqMan-PCR when mutation frequency in a given sample set was nearly 18%, and equal to sequencing only when the expected mutation frequency was at 83.5% (Figure 2 and Table S1).

**Figure 2:**
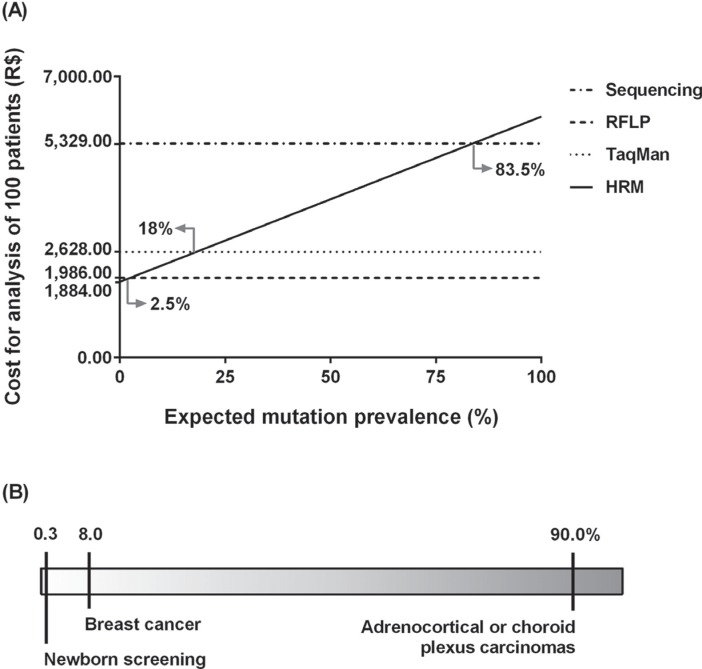
Cost variation of HRM analysis for screening of the *TP53*-p.R337H mutation according to estimated mutation prevalence. (A) Cost comparison of Sanger sequencing, TaqMan-PCR and PCR-RFLP. (B) Expected mutation prevalence scenario.

## Discussion

The mutant p.R337H founder allele has been found at a high frequency in patients diagnosed with tumors of the LFS/LFL spectrum (such as adrenocortical, choroid plexus and breast carcinomas) and also in the general population of Southern and Southeastern Brazil (Malkin *et al.*, 1990; Ribeiro *et al.*, 2001; Lalloo *et al.*, 2006; Achatz *et al.*, 2007; Palmero *et al.*, 2008; Garritano *et al.*, 2010; Seidinger *et al.*, 2011; Giacomazzi *et al.*, 2013; Cury *et al.*, 2014; Giacomazzi *et al.*, 2014). This scenario, of a highly prevalent germline mutation in a specific geographic region, is not unusual and has been described for many genetic disorders worldwide (Ewald *et al.*, 2011; Antczak *et al.*, 2013; Pinheiro *et al.*, 2013). In these situations, the use of robust (reliable) and at the same time affordable mutation detection techniques is essential. In the present study, we compared the performance characteristics and costs of four distinct genotyping techniques commonly used to detect the p.R337H founder mutation. Each method has its particular advantages and disadvantages, but genotyping results obtained with all four techniques were fully concordant, demonstrating that all of them can be reliably used for p.R337H detection. However, our results demonstrate that cost, turnaround and hands-on times can vary significantly with different methods, and a careful analysis should be done in determining which genotyping method is most adequate, depending on the infrastructure available, application and clinical scenario. It is important to emphasize that, as mentioned previously, we have not considered costs of acquisition of the equipments. We considered that there is significant diversity among institutions that could have influenced these costs (*i.e.* some institutions benefit from tax exemption incentives and others do not) and also recognize that Brazilian laboratories often use core facilities for diagnosis in their institutions, which eliminates the necessity of equipment acquisition.

In our analysis and with our laboratory setup, Sanger sequencing had the highest throughput when compared to other methods, but had the longest turnaround time and the highest cost (for single patient analysis it was 2.83 times higher compared to the least expensive procedure). Sanger sequencing requires advanced instrumentation, but the entire process is semi-automated, and both the laboratory protocols and result interpretation require significant hands-on dedication time. Despite these limitations, Sanger sequencing by capillary electrophoresis is still considered the gold standard in single gene mutation analysis in many centers and has been used in clinical genetic testing for many years. It is a robust, highly reproductive approach ideal for identification of mutations in a given DNA sequence, without necessity of previous interrogation of a specific mutation.

HRM, on the other hand is a mutation screening method, also widely used in clinical diagnostics, but it requires confirmation of genotype with a second method whenever a melting abnormality is identified. Several studies validated HRM for analysis of germline *TP53* mutations using different sample types and always demonstrating high sensitivity (81-100%) and specificity (83-99%) (Krypuy *et al.*, 2007; Bastien *et al.*, 2008; Garritano *et al.*, 2009). In the present study, sensitivity and sensibility of HRM analysis reached 100%, probably due to the use of high quality DNA obtained from leukocytes and to the short amplicon length used (87 bp). Compared to the other techniques assessed here, HRM had the lowest cost per patient, with a turnaround of nearly 5 hours, offering a convenient closed-tube method to assess the presence of single-base sequence variations. However, good laboratory practice recommends that amplicons with altered melting profiles be sequenced to identify which specific mutation or polymorphism is present, since different heterozygotes may produce similar melting curves. Thus, HRM is clearly suitable for mutation screening in populations with lower mutation prevalence (in the case of analyses directed to one single mutation) or with less disease-associated variants (in the case of mutation screening of an entire gene) (Li *et al.*, 2011).

In this study, we demonstrate that HRM analysis is cheaper than any of the other methods used when the predicted mutation prevalence in a given sample set is less than 2.5%, and less expensive than TaqMan-PCR or DNA sequencing when the estimated mutation prevalence reaches close to 18%. Thus, in Brazil, where the prevalence of p.R337H has been reported for several different sample sets, HRM would be an excellent strategy for mutation screening in the general population (mutation prevalence reported at 0.3% in a newborn screening program) and could also be considered in women with breast cancer (mutation prevalence up to 8%, depending on age at cancer diagnosis) along with other methods in this second group (Custodio *et al.*, 2013; Cury *et al.*, 2014; Giacomazzi *et al.*, 2014).

TaqMan-PCR, on the other hand, had the lowest turnaround and hands-on times in our study and has the great advantage of allowing simultaneous amplification and allelic discrimination in about 3 hours, without any further manual steps. However, as HRM, it had a low throughput in our study, due to the 48 wells real-time platform used and the need of performing reactions in duplicates, which both increase the overall time of analysis for large sample sets. TaqMan-PCR has a lower cost than Sanger sequencing and HRM when the expected mutation prevalence in the study population is above 18% (*i.e.* in some families with phenotypic criteria for Li-Fraumeni or Li-Fraumeni-like syndrome). For these situations and especially when results are needed quickly it is an excellent diagnostic approach.

Finally, PCR-RFLP showed reasonable costs and has the important advantage of minimal requirements in terms of investment in instrumentation. In addition, genotyping can be easily done by visualization of restriction fragments by gel electrophoresis, for which no specific software is needed. The most important disadvantage of PCR-RFLP, perhaps, is that it is a relatively time-consuming method, consisting of several sequential, and mostly not automated steps. In general, however, it is considered a simple, inexpensive and accurate method for genotyping, useful in small research studies and for laboratories that do not have advanced infrastructure or have limited financial resources.

In this study we compared performance, cost and turnaround time of Sanger sequencing, PCR-RFLP, TaqMan-PCR and HRM in the detection of a cancer predisposing founder mutation, *TP53*-p.R337H. This strategy, and results obtained here can be applied to other sequence variants associated with genetic disorders in high risk populations.

We conclude that multiple methodologies are suitable for the detection of *TP53*-p.R337H and genotyping results obtained in this study with these different strategies where fully concordant. The method of choice to be used in a given scenario will depend on the available laboratory infrastructure, acceptable time for result reporting and especially estimated mutation prevalence in the sample set to be analyzed.

## Supplementary Material

The following online material is available for this article:

Table S1 - Costs of *TP53*-p.R337H genotyping for 100 patients

Figure S1 - Results obtained with the different genotyping methodologies

This material is available as part of the online article from http://www.scielo.br/gmb
