# Supplementary figures and images for: Comparison of multiple genotyping methods for the identification of the cancer predisposing founder mutation p.R337H inTP53
Source: Genet Mol Biol. 2016 Jun 3;39(2):203–9. doi: 10.1590/1678-4685-GMB-2014-0351 (PMC4910550; doi:10.1590/1678-4685-GMB-2014-0351)

(A)

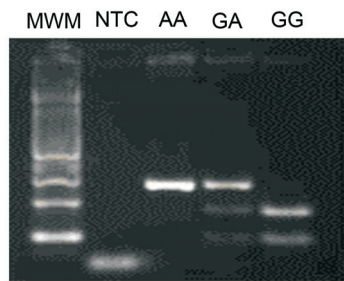

(B)

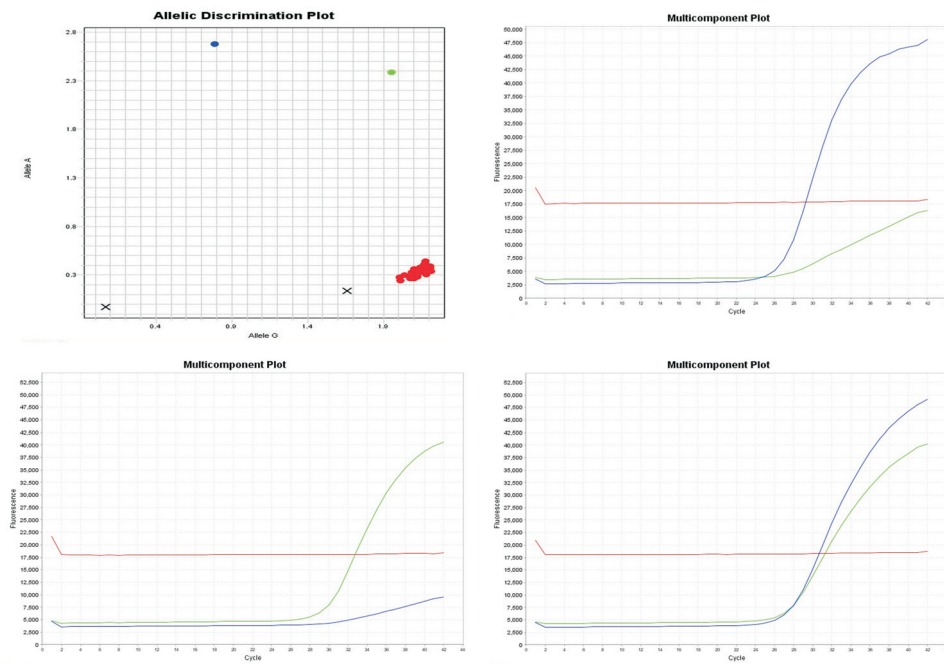

(C)

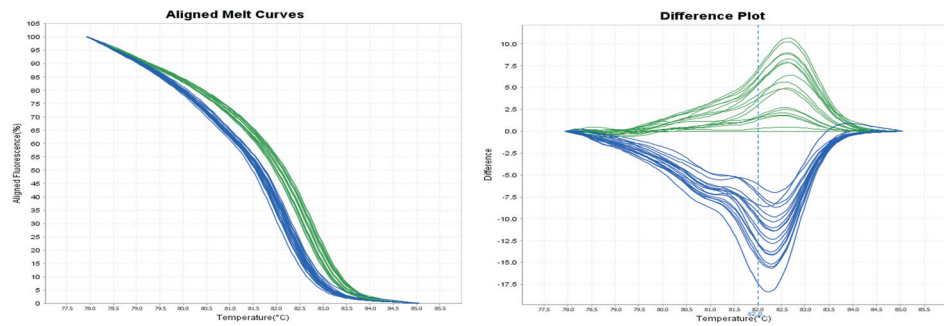

(D)

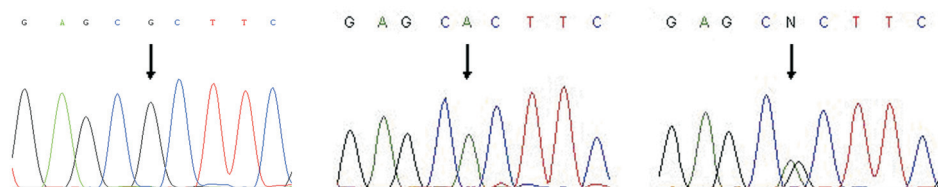

Figure S1 - Results obtained with the different genotyping methodologies

Supplement: Supplementary file 1 [file 1415-4757-gmb-1678-4685-GMB-2014-0351-Suppl02.pdf]
