# Supplementary material for: Comparison of multiple genotyping methods for the identification of the cancer predisposing founder mutation p.R337H inTP53
Source: Genet Mol Biol. 2016 Jun 3;39(2):203–9. doi: 10.1590/1678-4685-GMB-2014-0351 (PMC4910550; doi:10.1590/1678-4685-GMB-2014-0351)
Supplement: Supplementary file 2 [file 1415-4757-gmb-1678-4685-GMB-2014-0351-Suppl01.pdf]

Table S1 - Costs of *TP53*-p.R337H genotyping for 100 patients according to expected mutation prevalence.

**Method**	**Costs in different mutation prevalence scenarios (R$)**			
	**0.3%**	**2.5 %**	**18.1%**	**83.7%**
**DNA Sequencing**	5,329.00	5.329.00	5,329.00	**5,329.00**
**PCR-RFLP**	1,986.00	**1,986.00**	1,986.00	1,986.00
**TaqMan-PCR**	2,628.00	2,628.00	**2,628.00**	2,628.00
**HRM followed by DNA sequencing[a]**	**1,896.35**	**1,986.92**	**2,629.17**	**5,329.92**

[a] sequencing from same DNA sample (no new DNA extraction) - cost of R$ 41.17 for patient
